# Amplifying the Voices of Indigenous Elders through Community Arts and Narrative Inquiry: Stories of Oppression, Psychosocial Suffering, and Survival

**DOI:** 10.1002/ajcp.12367

**Published:** 2019-07-31

**Authors:** Amy F. Quayle, Christopher C. Sonn

**Affiliations:** ^1^ Institute of Health and Sport Victoria University Melbourne Vic. Australia

**Keywords:** Aboriginal storytelling, Narrative inquiry, Community psychology, Community arts and cultural development, Decolonization

## Abstract

Researchers and practitioners in community psychology have an important role to play in supporting decolonial work including promoting opportunities for reclamation, healing, and acknowledgment of history. In this article, we discuss research undertaken alongside a community arts and cultural development project that sought to support Aboriginal people in Western Australian to create an archive of their stories for current and future generations; stories that could serve as resources for healing, reclamation, and for examining a painful and unjust past. Narrative approaches have been promoted in community psychology to advance empowerment research and practice alongside marginalized, excluded, and minoritized groups. We report on findings from a critical narrative inquiry of the stories shared through the project and in conversational interviews with four Noongar Elders to explicate the history and ongoing legacy of racialized oppression in their lives as well as cultural continuity and survival evident in the stories. Community researchers and practitioners can play a role in amplifying those stories as part of the co‐intentional work of decolonization.

## Highlights

- Community psychology can contribute to healing and cultural renewal for indigenous communities.
- Storytelling through community arts practice is used to witness Elder stories.
- Narrative inquiry shows the ongoing effects of colonisation and coloniality.
- Narrative inquiry shows the various ways people resist and survive oppression.
- Decolonial approaches are vital to the goals of critical community psychology.

## Introduction

As a settler colonial nation, the systematic dispossession of Indigenous people through frontier violence and assimilationist policies, practices, and discourses has marked Australia's history. Like the settler colonial experience in other parts of the world, the colonizers have not left and remain institutionally and culturally dominant, and the Indigenous population constitutes a numerical minority of the population. Australia's Indigenous people are two distinct cultural groups, Aboriginal and Torres Strait Islander peoples, made up of many diverse nations. The 2016 census estimated an Indigenous population of 798,400 constituting 3.3% of the total Australian population; 91% identified as Aboriginal, 5% as Torres Strait Islander, and 4% as both Aboriginal and Torres Strait Islander (Australian Bureau of Statistics, 2018).

Similar to the colonial experience elsewhere, the process of colonization has had long‐lasting impacts for Indigenous peoples, culture, and communities. As noted by Krieg (2009), “colonization was not a moment – but is an ongoing experience with multiple persistent contemporary traumatizing events continuing to impact daily on Aboriginal families and communities” (p. 30). For example, the intergenerational impacts of the systematic and forcible removal of children of Australian Aboriginal and Torres Strait Islander descent from their families in what came to be known as the Stolen Generations, continues to be felt (Aboriginal and Torres Strait Islander Healing Foundation [ATSIHF], 2017).

### The Stolen Generations

The Stolen Generations refers to those children of Aboriginal and Torres Strait Islander descent systematically removed from their families under various government policies rooted in assimilationist ideology. For example, in Western Australia (WA), advocating segregation and the strict implementation of the *1905 Act* (WA), “Chief Protector of Aborigines,” A. O. Neville, introduced the “Native Settlement Scheme” which saw Aboriginal children of mixed descent “physically separated from their families on the settlements, receive a European education, be trained in domestic and stock work and then sent out to approved work situations” (Human Rights and Equal Opportunity Commission [HREOC], 1997, p. 91). It was believed that this process would result in the acceptance of “half‐caste” children by non‐Indigenous people as well as the loss of identification with their Aboriginality (Haebich, 2000). Similar policies and practices were enacted in other settler colonial nations such as the Residential School system in Canada (Archibald, 2006).

The National Inquiry into the Separation of Aboriginal and Torres Strait Islander Children from their Families (i.e., the Bringing Them Home report) (HREOC, 1997), reported that from 1910 to 1970 between one in 10 and three in 10 Indigenous children were forcibly removed from their families and communities, and all Indigenous families have been affected. The report concluded that policies and practices of child removal amounted to genocide as defined in the United Nations Convention on the Prevention and Punishment of the Crime of Genocide because “When a child was forcibly removed that child's entire community lost, often permanently, its chance to perpetuate itself in that child” (Human Rights and Equal Opportunity Commission, 1997, p. 190).

The ongoing, intergenerational effects of these policies and practices continue to be felt. The Australian institute of Health and Welfare (AIHW, 2018) recently released a report examining health status, health risk factors, cultural factors, and socioeconomic indicators of surviving members of the Stolen Generations and their descendants. Based on census data, it was reported that surviving members of the Stolen Generations (estimated to be 20,900 individuals) were more likely to: have been incarcerated in the last five years, have been formally charged by police in their lifetime, have government payments as their main income source, not be a home owner, and have poorer general health. In comparison with a reference group of Indigenous people (18 years and over) who had not been removed or had relatives removed, descendants also consistently fared worse on a range of health, socioeconomic, and cultural indicators (Australian Institute of Health and Welfare, 2018).

Indigenous peoples have utilized the language of trauma to understand and contextualize their experiences (e.g., ATSIHF, 2017; Archibald, 2006; Krieg, 2009). In Australia, Atkinson (2002) described trauma trails, noting how collective experiences of trauma, seep “slowly and insidiously into the fabric and soul of relations and beliefs of people as community” (p. 53). The effects of trauma for Indigenous peoples in Australia echoes work in other settler colonial contexts. Duran, Duran, Brave Heart, and Yellow Horse‐Davis (1998) used the notion of a “soul wound” to describe the destructive legacy of histories of colonization and the continued impacts on the psychology of Indigenous peoples in North America. The concept of historical trauma has also been used to name and interpret colonial histories and the ongoing legacies of these histories for the colonized. Brave Heart (2003) described historical trauma as the “cumulative emotional and psychological wounding, over the lifespan and across generations, emanating from massive group trauma experiences” (p. 7). These concepts have been applied to understand and contextualize issues of suicide, violence, and substance misuse within Indigenous communities (e.g., Atkinson, 2002; Brave Heart, 2003; Duran et al., 1998). Importantly, Denham (2008) has asserted the need to be cognizant of the various responses to historical trauma which can include expressions of suffering, but also, and significantly, expressions of resilience, resistance, and survivance (Vizenor, 2008).

#### Toward Reclamation and Healing

The Bringing Them Home report outlined 54 recommendations to support healing and reconciliation for the Stolen Generations, their families, and the Australian public more broadly including acknowledgment and formal apology. A Government apology was finally passed by Parliament on February 13, 2008. Twenty years since the release of the report, the Aboriginal and Torres Strait Islander Healing Foundation (2017) highlighted the continuing effects of the Stolen Generations and noted that the great majority of recommendations have not been implemented. Their action plan for healing listed important areas for action including healing intergenerational trauma and creating an environment for change which necessitates “a truthful examination of a painful and unjust past” (Aboriginal and Torres Strait Islander Healing Foundation, 2017, p. 46). Researchers and practitioners in community psychology have an important role to play in supporting the implementation of programs that align with such an action plan. Given that research and practice involving Indigenous peoples has been described as exploitative, dehumanizing, and disempowering (Dudgeon & Walker, 2015; Smith, 1999), there is a need to decolonize research (and practice) with Indigenous peoples.

### Decolonizing Research and Practice with Indigenous Peoples

The problematic history of research and practice with Indigenous peoples has been increasingly recognized within psychology and other disciplines. Glover, Dudgeon, and Huygens (2010) discussed the roles of psychologists in decolonization as deconstructing and critiquing dominance and injustice, learning to practice in the presence of history, affirming Indigenous authority, expertise and self‐determination, and listening, protesting, and advocating. Dudgeon and Walker (2015) outlined several strategies for decolonizing psychology including challenging mainstream psychological conceptions through the production of new knowledges and discourses, acknowledging power relations and white privilege, a focus on “Indigenous‐led strategies, solutions, tools, and methods to support critical reflexivity” (p. 292), promoting cultural resilience and recognizing and supporting Indigenous spirituality.

Central to the calls for decolonizing research is the imperative to center Indigenous knowledge and experience. Those who work in this space, both within and beyond community psychology, have drawn from various areas of critical scholarship to problematize dominant modes of knowledge production and expand the dialogue of voices and the ecology of knowledge (Santos, 2014). Creating settings for storytelling and counter‐storytelling has therefore been a central strategy for decolonization (Montero, Sonn, & Burton, 2017; Smith, 1999; Sonn, Stevens, & Duncan, 2013; Zavala, 2016).

Storytelling as methodology is central to learning and understanding colonial histories and legacies but also in constructing identities in the present (Kovach, 2009; Smith, 1999). In her seminal writing on decolonizing methodologies, Smith (1999) outlined 25 projects for Indigenous peoples including claiming testimonies, storytelling, celebrating survival/survivance, remembering, connecting, revitalizing, and regenerating. Kirmayer, Dandeneau, Marshall, Phillips, and Williamson (2011) noted that collective forms of narrative afford people the opportunity to make sense of their experience, construct a valued identity, and ensure the continuity and vitality of a community or a people. Gonzalez, Simard, Baker‐Demaray, and Eyes (2014) emphasized the need to recognize the many stories Indigenous peoples have to tell: “Stories about loss. Stories about suffering. Stories about pain. Stories of genocide and destruction. But there are also stories of survival. Stories about resilience. Stories about pride… stories about healing” (p. 32). The authors expressed that as Indigenous peoples they “want to talk about who [they] are, where [they] come from, and the great trauma that [they] have survived and continue to face so that [they] can learn to heal together” (Gonzalez et al., p. 32). These different authors have highlighted that while many Aboriginal people are well aware of the oppressive forces at work in their everyday lives, they need opportunities to tell their stories. In this article, we describe a community arts and cultural development (CACD) project that sought to provide such opportunities. We report on the first author's doctoral research (Quayle, 2017), which used narrative inquiry to analyze and interpret the stories produced through the project and in conversational interviews with Elders.

## Method

### Context: Rekindling Stories on Country

This research was conducted in partnership with Community Arts Network (CAN) of WA, a CACD organization working with Noongar communities across the Wheatbelt region of WA. CACD involves collaborative work between artist‐organizers and other community members “to express identity, concerns and aspirations through the arts and communications media. It is a process that simultaneously builds individual mastery and collective cultural capacity while contributing to positive social change” (Goldbard, 2006, p. 20). The Wheatbelt region is one of nine regions of WA, covering an area of 154, 862 square kilometers across 43 local government authorities (Department of Regional Development, 2014). Some of the towns in the Wheatbelt have a relatively large Indigenous population (e.g., closer to 10 percent vs. 3.3 per cent nationally), with most identifying as Aboriginal and specifically Noongar.

Noongar people are the traditional custodians of the southwest corner of WA, and one of the largest Aboriginal cultural blocks in Australia, made up of 14 different language groups. There are approximately 30,000 Noongar people living in the southwest of WA (Government of Western Australia, 2017). *Moort* (family), *boodja* (land), and *Kaartdijin* (knowledge) are central to Noongar culture and identity (Collard, Harben, & van den Berg, 2004). Over years, CAN has carefully developed relationships and trust with Noongar communities across the Wheatbelt and delivered several successful projects culminating in the Rekindling Stories on Country strategy. Central to Rekindling Stories on Country is the creation of opportunities for intergenerational storytelling and cultural transmission and a platform for Noongar stories to be shared with a broader audience (Community Arts Network Western Australia, 2014). This strategy followed requests from Noongar people who told CAN staff, “Our young people are dying; they need their culture” (Community Arts Network Western Australia, 2014, p. 73).

Bush Babies was one project delivered as part of this broader strategy across a number of Western Australian towns. The Bush Babies project began with the simple idea of honoring Noongar Bush Babies and the midwives who delivered them. As noted by Community Arts Network Western Australia, (n.d):

These stories are from a time when Aboriginal people were not permitted to live in towns or cities, and definitely not permitted to give birth in a hospital. Instead, Bush Babies were born in reserves, missions and on the outskirts of town in tents, makeshift shelters and under the stars.(p. 2)

Reserves and missions were places that Aboriginal people were forcibly relocated to, with missions typically run by churches and reserves run by government and sometimes churches (Australian Institute for Aboriginal and Torres Strait Islander Studies, [AIATSIS], 2016).

Since its beginnings in 2010, Bush Babies has been delivered in numerous towns across the Wheatbelt taking different shape in response to the stated aspirations of Noongar people in each of the towns. The central thread across each of the projects has been capturing, celebrating, archiving, and elevating stories of Noongar people; stories about being born in the bush, and/or growing up on reserves, in missions, and on the fringes of towns, as well as stories about what it means to be Noongar.

The Bush Babies project of focus in the research involved various elements including intergenerational storytelling workshops with young people and Elders, photographing Elders on country and the recording of their Bush Babies stories, Storylines workshops, and the Honouring our Elders portrait project and exhibitions. The intergenerational storytelling workshops involved a number of Elders sharing stories about their lives with young Aboriginal media studies students, who were then supported to create short videos using photographs to go along with the edited stories. The Storylines workshops were conducted in local primary and secondary schools. Students were encouraged to invite their Elders along to participate in the workshops where they were introduced to the Storylines database, “an online archive for the State Library's digitised heritage collections relating to Aboriginal history in Western Australia” (State Library of Western Australia, 2016).. The Honouring our Elders portrait project developed following a local exhibition of photographs that inspired a local non‐Indigenous artist to seek permission to paint the portrait of one of the Elders. This grew into a larger project involving 12 local artists, two of whom were Aboriginal, painting the portraits of 16 local Elders. These portraits were later exhibited locally and at the Perth Museum and toured across the state. During exhibitions, the portraits were accompanied by snippets of the Elders’ Bush Baby stories including the short videos that were produced by students (For more on the Bush Babies project see Community Arts Network Western Australia, 2014).

### Researcher Positioning

As non‐Indigenous researchers, we are both outsiders to this community. The first author is a white Australian woman who lives in Melbourne on the country of the Woiwurrung and Boonwurrung of the Kulin people. She became involved with the work of CAN as part of her Masters research. The second author identifies as a black man from South Africa who has been a critical friend of the organization over many years taking on various roles including research, evaluation, and education about race, whiteness, and racism.

We are both positioned differently in relation to the colonial matrix of power, that is, intersections of race/gender/whiteness, and we continue to wrestle with the complexities of this in our everyday lives, research, and practice. Standing on the sidelines is not an option, we must engage with racism and whiteness and figure out ways to enact relational, ethical epistemologies in full recognition of the responsibility we have as settlers and academics in the co‐intentional work of decolonization (Glover et al., 2010). In undertaking such work, we recognize the importance of remaining vigilant to dynamics of power afforded by our various social group memberships and how these are expressed in everyday settings. Reyes Cruz and Sonn (2011) advocated a decolonizing standpoint in research and action. This orientation requires that in community engaged work, attention is given to social locations/positionalities because these have implications for how we are perceived, our own subjectivities and intersubjective relations.

While initially the research set out to examine the role of CACD within and beyond Aboriginal communities, the focus shifted to amplifying the stories shared by Noongar Elders through the project. This decision was part of a reflexive process driven by the intention to be responsive to the wishes of Noongar people as part of enacting a respectful and ethical relationship. It was about centering their voices, knowledge, and experiences but also reflected recognition of stories as a means of exploring dynamics of oppression and as sites of resistance, reclamation, and healing (Sonn et al., 2013). The research sought to explore the question: What are the narratives through which Noongar people involved in the Bush Babies project give meaning to their past, present, and future and what are the key themes in these stories?

### Exploring the Stories through Constructionist Narrative Inquiry

In this research, we sought to extend the platform for Aboriginal storytelling on country by analyzing the stories shared as part of the project and in conversational interviews with four participating Elders. A critical, constructionist approach to narrative inquiry was chosen because its tenets align with the decolonial agenda. Sparkes and Smith (2013) noted that within a narrative constructionist approach, narratives are understood as “forms of social action through which human life and our sense of self are constructed, performed and enacted” (p. 299). From this view, “selves” (or identities), “memories,” and “emotions” are understood as “constituted through storytelling and shared resources” (p. 299).

Smith and Sparkes (2008) discussed the storied resource perspective as one narrative approach to understanding selves and identities. Within this approach, the analytical lens is turned to the “socially situated production of identity” (Smith & Sparkes, 2008, p. 17), where narrative is conceived as “a form of social practice in which individuals draw from a cultural repertoire of available stories larger than themselves that they then assemble into personal stories” (p. 19). These personal stories “cannot be extricated from the social” (Smith & Sparkes, 2008, p. 20) and power relations; instead, they are understood as an “achievement by persons in relationships, employing resources held in common with other people” (p. 20).

#### Participants and Data Collection

To develop respectful relationships and gain support for the research from key Aboriginal community representatives (AIATSIS, 2012), the first phase of the research involved several fieldwork visits beginning in November 2013 by the first author. CAN staff, including two local Noongar people, played a central role as cultural consultants acting as an informal reference group throughout the research process (Walker, Schultz, & Sonn, 2014). During this phase, the first author attended various workshops, launches, a community festival, participated in Bush Babies and other related project activities, and had informal conversations with Elders, artists, and facilitators, Aboriginal and/or Torres Strait Islander young people, and other community members involved in CAN projects. This was important in developing an understanding of the context, relationship building, and in the iterative and generative development of the research focus.

Recruitment of research participants for conversational interviews began in July 2014 following the official launch of the Honouring our Elders Portrait Project Exhibition in Perth. The official launch represented the culmination of the project; this was considered an opportune time to speak about the meaning and significance of the project for participants. The trust that the agency had built up with Noongar people was central to the willingness of participants to speak with the researcher. Noongar staff at the CAN office encouraged Elders to come and “have a yarn” (i.e., an informal conversation) about their Bush Baby story and the Bush Baby project at the CAN office which had become a meeting place or drop in center for Noongar people in the town. We also visited Elders in their homes to share information about the research and invite them to come down to the office.

It was important to be flexible and patient. During this phase, the first author had many informal conversations with CAN staff who shared their reflections on the project and the significance of Rekindling Stories on Country, their own healing journeys, and discussed issues facing the Noongar community and Aboriginal people more broadly. We also reflected upon and discussed the key themes evident in the stories the Elders were sharing in the interviews and informal conversations. Sitting around and yarning was part of the important work of building relationships, waiting until people were ready to share, and being open and transparent.

Bennett, Zubrzycki, and Bacon (2011) emphasized the importance of "building the relationship in environments where the person feels comfortable; being aware of community protocols regarding with whom to consult; working alongside people; not going straight down to business or being outcome and process driven; providing choices about with who they might want to work…; giving information and working from a position of humility and dealing with people with dignity"(p. 29).

These practices are important aspects of cultural safety (Kickett‐Tucker, Bessarib, Coffin, & Wright, 2017).

Four Noongar Elders who had participated in the Bush Babies project were interviewed: two males and two females all in their late 60s. Two of the Elders were married and were interviewed together. Each of the Elders interviewed had their portrait painted for the project. Three of the four Elders participated in the storytelling workshop at the High School and their stories were recorded, with many made into digital stories. Each of the Elders had been involved in different CAN projects. Three of the four Elders interviewed indicated that they were members of the Stolen Generations. The other Elder grew up on a reserve on the outskirts of town.

Initial interviews took place at the CAN office with a Noongar staff member who played an important role in creating a relaxed and safe interview environment. Seemingly small assurances such as stating to Elders that we were, “just having a yarn,” helped to set the climate of safety. Bessarab and Ng'andu (2010) have described yarning as “an Indigenous cultural form of conversation” (p. 37) and suggested that yarning is “conducive to an Indigenous way of doing things; its strength is in the cultural security that it creates for Indigenous people participating in research” (p. 47). The conversational style of interviews enabled Elders to lead the direction of the topics discussed in interviews, thereby reshaping the research focus.

Participants consented to their participation in conversational interviews and for these interviews to be digitally recorded and transcribed verbatim. As per the ethics approval, pseudonyms were used to protect the confidentiality of research participants. In retrospect however, this is something we should have negotiated with the Elders without assuming that they would want their stories anonymized.

The interviews, which ranged from one hour to two and a half hours long became an opportunity to bear witness to the Elders’ stories and memories of lives directly impacted by assimilationist policies, practices, and discourses and the legacy of this in the present. In bearing witness to these stories, the first author, as someone who had expressed a willingness and interest in listening to and hearing these stories as a means of interrogating our history and its continuities in the present, became accountable to the Elders.

In May 2015, approximately 10 months after the initial interviews and the official launch of the Bush Babies project, the first author travelled back to the town to share with Elders the themes that had been identified in the process of transcription and preliminary analysis. These interviews also provided an opportunity to have a more focused discussion in these key areas. On this visit, the first author was able to speak with three of the four Elders again.

**Figure 1:**
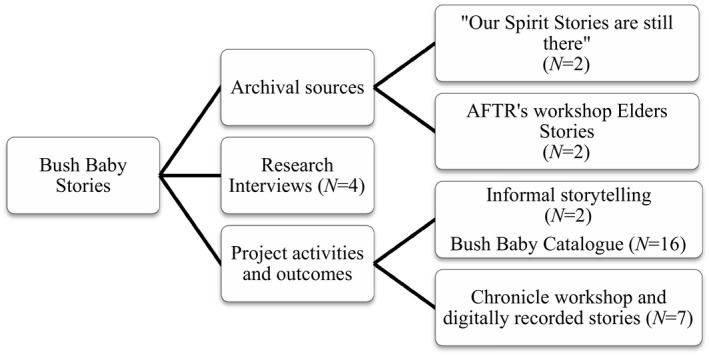
Bush Baby data sources

The research also made use of various archival sources including Bush Baby stories recorded as part of project activities both informally and as part of the intergenerational workshop at the high school and a local community festival. Snippets of the stories of all 16 Elders were also included as part of the Bush Babies catalogue. Archival resources produced as part of related CAN projects, which provide an archive of Elders’ stories, were also included as Bush Baby stories. Some of the Elders came to the interviews prepared with documents, photographs, and/or newspaper articles; they wanted to share aspects of their life stories. Making use of this material was considered particularly important given that Aboriginal people are often burdened with the expectation of telling their stories and sharing their knowledge multiple times. Figure 1 provides details of the sources of Bush Baby stories.

#### Data Analysis Framework

In analyzing the Elders’ personal stories, the aim was to identify the shared community narratives evident across the Bush Baby stories. The personal stories were conceptualized as providing insights into the resources Noongar people use to make sense of past, present, and future, and in constructing what it means to be Noongar. As well as highlighting the symbolic resources available at a community level, these personal stories were also understood in relation to broader cultural narratives (Rappaport, 2000). In this regard, the Elders’ stories were conceptualized as counter‐stories (Solórzano & Yosso, 2002).

It was important to carefully navigate how we interpreted and represented those stories. Chase (2005) discussed three voices used by narrative researchers: the authoritative voice, the supportive voice, and the interactive voice. The supportive voice “pushes the narrator's voice into the limelight” (p. 665). In presenting the analysis, the aim was to stay close to the stories and keep the Elders’ stories central. This involved keeping the researcher's voice to a minimum, guiding the reader, and making connections between stories as well as with relevant scholarly literature.

#### Data Analysis Process

The recorded Bush Babies stories which included conversational interviews with four Elders, the stories digitally recorded as part of project activities, and those Bush Baby stories recorded as part of related CAN projects, were transcribed. As discussed by Crossley (2007), “meaning is not just ‘transparently’ available within an interview, a transcript or an autobiographical script. It has to be achieved through a process of interpretation and engagement with the text” (p. 147). Recordings were listened to multiple times throughout the process of transcription. Transcriptions were read and re‐read repeatedly to develop a general sense of key emerging themes.

After multiple readings, the first author produced initial summaries for each of the Elders who shared their Bush Baby story/stories. These summaries outlined significant life events (e.g., taken to mission as a young child), key themes or emphasis in their storytelling, and similarities and/or differences across settings (i.e., interviews vs. storytelling workshop). The preliminary coding process involved reading the transcripts line‐by‐line taking note of sections of the text relevant to the research questions. A process of open coding (Miles & Huberman, 1994) for each of the individual transcripts then took place. The focus at this stage was on the individual Bush Baby stories. Based on this initial coding, a coding scheme was developed. Examples of codes included “hard but happy lives,” “family feuding,” “concern for young people,” “strong extended family on reserves,” “loss of culture,” “cultural continuity/survival.”

The coding scheme that was developed in the first stage was then used in rereading the transcripts, searching for statements that fit into the emerging categories, but also being open to any additional codes. This stage therefore involved looking across the transcripts to identify the shared aspects of the stories being told, that is, the community narratives evident across the stories. The coding scheme was developed and refined as the analysis progressed. These themes reflected shared aspects of the Elders’ stories; the shared narrative resources that they were drawing on in constructing personal stories.

Following these first two stages, the aim was to become more analytical, and look for patterns and explanation in the codes and organize the codes into themes (Miles & Huberman, 1994). The aim was to create a coherent and persuasive narrative based on the emerging themes (Lyons, 2007). It was important that this narrative include the many stories that were told—stories of hardship, loss, despair, but also, stories of family, cultural continuity, and survival. This process involved analyst triangulation through reflective discussions and feedback on emerging themes with two research advisors. The stories were ultimately organized into community narratives relating to key themes across their storytelling. Critical narrative analysis generates “a situated, partial and warrantable psychosocial ‘knowledge’” (Emerson & Frosh, 2004, p. 10). Here, the situated knowledge of Noongar Elders was made central.

### Research Findings

The aim of our inquiry was to examine the stories of “Bush Babies” to understand the historical and contemporary experiences of racialized oppression, its impacts upon individuals and communities, and how people resist and survive oppression, historically and in contemporary times. Three broad community narratives were identified that provided important insights into culture and context and its profound effects on individuals’ lives. Through the project the Elders were narrating circuits of dispossession, past and present, the consequences of dispossession for individuals, communities and across generations, but also cultural continuity and survival. In the following sections, we discuss each of the narratives and their sub‐themes, drawing on illustrative excerpts. Pseudonyms have been used for interview participants. Pseudonyms have not been used for publicly available archival material.

### Narrating Circuits of Dispossession

The first community narrative showed the circuits and accumulation of dispossession for Aboriginal people, past and present and has been reported elsewhere (see Quayle, Sonn, & van den Eynde, 2016). The Elders narrated how “life was put on [them],” but they also emphasized “it's still going on.”

#### The Collective Remembering of Oppression: “Life was put on us”

The stories shared by Elders included references to mechanisms of control that were central to colonialism historically (Fanon, 2004; Moane, 2011), including the ideology of “race” that constructed “blackfellas as the outcast”, the use of fear and violence “to keep them down” and the systematic fragmentation of families, communities and culture so they were not able to learn Noongar culture or be a family. As noted by Caroline as part of an intergenerational storytelling workshop:

…they made to be as a as a different type of, as a different person to everyone else in society because of um the colour of our skin and um “cause how, who we are, Aboriginal people. …. We was taken away from our parents and put in the missions …reserves outside of towns, near dumps, near the bush where they segregate Aboriginal people away, out of sight because of their status in life, being a lower class people. But it's not right really.”

#### Narrating the Continuity: “It's still going on”

In their storytelling, the Elders’ named the continued experience of structural, cultural, and interpersonal forms of violence in the everyday lives of Aboriginal people—the fact that they are “still underneath” (Mick). They named ongoing disparities in health, wealth and opportunity, the silencing of the history of dispossession and its implications in the present, and shared experiences of everyday racism and disregard. For example, Caroline emphasized that Aboriginal people have still “got nothing” and are “still controlled”:

We're still – our life is still struggling. We're still trying to establish ourselves… Never got near the establishin’ side of our life yet. We're still – There's no freedom there. We're still controlled, you know, well there's Homes West, Government, Prime Ministers, Government, all different people of authority has control of us, you know, “cause we got nothing. We'll never have nothing.”(Interview)

Mick also highlighted the continuing disparities between Aboriginal and non‐Aboriginal lives:

You look around and you see all these other big flash things all going up every day yet we still got nothing. Still got nothing *(laugh),* richest country in the world yet we're still living back in the 60s, you might say, still got nothing.(Interview)

### The Consequences of Dispossession: “This is Where it all Stems From”

The second community narrative, “This is where it all stems from” provided insights into psychosocial suffering, then and now, as narrated by the Elders. Psychosocial suffering is an approach that recognizes the generation of psychological distress, reflected in intrapsychic and relational wounds, in conditions of structural violence (Frost & Hoggett, 2008). Specifically, the stories showed the psychic harm carried by individuals (“It's burnt in our brains”), the disruption to culture and community (“No‐one listens anymore”), and the effects across generations (“Our kids are carrying what we been through”).

#### Psychic Harm: “It's burnt in our brains how we was treated like animals”

Mick captured the psychic harm of oppression using the words “branded,” “burnt,” and “locked in our brains,” and referred to how Aboriginal people have been treated “like animals.”

Mick: Yeah I thought to myself you wouldn't have a clue to live in a black man's shoe, you know shoes, walking their footsteps, or whatever, you wouldn't have a clue to know what it's like lookin’ at it from our side of the fence. It wouldn't even enter their brains like its locked in our brains you know, it got burnt in our brain, you might say, we got branded on our brains …It's still there, you know how we was treated, like animals, you might say.(Interview)

Mick's comment highlights the need to understand the constitution of Aboriginal subjectivities within a context of coloniality where Aboriginal people have been relegated a sub‐human status—where the cultural tools available for appropriation are psychologically damaging to Aboriginal subjectivities (Atkinson, 2002; Gonzalez et al., 2014).

#### Community Fragmentation: “No‐one listens anymore”

The Elders spoke of the forced erosion and loss of cultural knowledge and language stemming from dispossession, and the psychosocial implications for communities. For example, Caroline described how assimilation meant that their parents were not allowed to pass on cultural knowledge because they had “to adapt to white people's lives” *(Intergenerational storytelling workshop)*. All the Elders who were interviewed were members of the Stolen Generations, or their parents, grandparents, or great‐grandparents were, and so they each expressed that they did not know much in culture or language, and this was experienced as a loss. Enid said, “I don't know my Noongar language,” and “I didn't learn much in culture, but I knew about the respect, storytelling and (*pause*) yeah, maybe it's left to us to the do the writing”.(Interview)

In the second interview with Mick, he expressed a sense of despair about the issues facing the Noongar community, asserting: “dysfunction, everything dysfunction.” Mick understood this “dysfunction” as a consequence of past policies and practices that were explicitly aimed at the removal of culture, and thus, the destruction of traditional roles, structures, and knowledge. Mick explained there is “…no guidelines, no discipline.” He was speaking about the loss of respect for Elders but also pointed to the negative influence of technology on young people.

Mick: Well everything's, everything's lost now, too many things been happening too much technology coming out now and taking the young fellas away from everything …. When my old grandfather he ah, I'll just read ya what he said. He died … 1968 he died… yeah so not long before he died he remarked, “I'm worried about my people and what will become of them, as they won't listen to advice anymore.”

Interviewer: Is that what you feel as well?

Mick: Worse, just like he said, they won't listen no more. It's all yeah it's what um, old Mr. Neville said, he wanted a white Australia…it's trying to be a white Australia and try to rub out all stuff Aboriginal….(Interview)

#### Intergenerational Impacts: “Our kids are carrying what we been through”

The intergenerational impacts of this history were also evident in the Elders storytelling. Caroline explained to a small group of Aboriginal students, the “government thought they were doing a good job…. a lot of the families…they're still carrying it—they'll always be carrying it.” Caroline described the devastating impact that policies of child removal have had on the family unit and Aboriginal communities more broadly. In the example below, Caroline described the pain of seeing her grandchildren not having their parents, and of her children not being with their children.

I pray for my kids because they all led astray, “cause they going through their trauma too, most probably because we been, both parents been in the mission and now the childrens [sic] are feeling our trauma and I praying for them too because I know they're weak, … they get sidetracked.”(Intergenerational storytelling workshop)

Testament to the intergenerational impacts, Caroline feared that the children of those who were taken to missions could be “our lost generation.”

This narrative brought into sharp focus the devastating consequences for the Stolen Generations with the impacts of these policies and practices understood as insidious and cumulative and transmitted across generations. Twenty years since the Bringing them home report (Human Rights and Equal Opportunity Commission, 1997), the Elders’ stories therefore showed the devastating consequences and reverberations, which continue in the present.

### Cultural Continuity and Survival: “We Survived, we're still here”

While the second broad community narrative highlighted the removal of culture and language and the destructive effects of this history for connectedness and relationships, the Elders’ stories also highlighted the continuity of cultural knowledge and practice, thus showing the dialectic of oppression and resistance (Fine, 2014) or survivance (Vizenor, 2008). In particular, the stories shared by Elders pointed to the ongoing significance of Aboriginal knowledge reflected in *moort* (family and kinship), *boodjar* (country), and *katitjin* (cultural knowledge and practice) (Collard et al., 2004).

#### Cultural Knowledge and Enactment: “Culture is not dead”

Through their stories, the Elders discussed the significance of cultural understandings and practices that enabled them to survive on the fringes where they experienced grinding poverty, as well as how they found spaces to express their cultural identity and engage in cultural practice. The bush was identified as a space of freedom and healing. For Janet, going out bush was about:

…getting back to their original I suppose you could say identity because a lot of them seemed to think once they got into town, with the problems that were existing in towns and the racism that existed in most of the country towns around where I grew up, it was better to be in the country, it was better to be in the bush because out there you had a freedom to express yourself and a freedom to work with the people who accepted the Noongar people so it was really good.(Story recorded as part of a related CAN project)

The stories thus highlighted how Aboriginal/Noongar people created alternative settings where they were able to protect, maintain, and renew valued aspects of identity and culture that were devalued within the larger society (Sonn & Fisher, 1998). These alternative settings—out bush, on reserves—were spaces where Noongar people could speak language and practice culture away from non‐Indigenous people/society and thus engage in practices central to the continuity of valued identities and ways of living. Revel, who grew up on a reserve on the outskirts of town, emphasized the knowledge he learnt from his “old people” on country, and declared “Culture is not dead, through my life I've been through it I know what's it's like” *(Story recorded as part of a related CAN project)*.

#### Connection with Country: “It's still Noongar country”

The continuing significance of country for Noongar people and the act of going back to country with their children and grandchildren was also captured in the Elders’ storytelling. In sharing her story, Janet emphasized the connection between kinship, country, and the cultural knowledge that comes with it as she described her family's “Noongar run” or *Moort Boodja* (family country). She explained, it was “always a symbol of kinship…for our Noongar people. … that run … that was always special, it was always a symbol of family, kinship, getting together, new birth, all the birthing places along that river, along that road and most of the camp sites along there”. For Janet, going back to country was connected to memories of her family.

…to me going back to those places today and taking my family back, you know, it makes you feel good because it's your country, you feel like you haven't lost it, It's still Noongar country and there's a lot of stories relating to all those countries.(Story recorded as part of a related CAN project)

Similarly, Enid expressed the significance of the old reserve: “I mean the reserve up here's special” *(Interview)*. Enid described her strong connection with the town, because it is the country of her parents, grandparents, ancestors and she can “feel their presence” when she walks on the reserve.

#### Kinship: “We are all connected”

The Elders’ stories about family and kinship communicated the significant role that extended family played in mediating the impacts of oppression and thus of surviving on the fringes. Enid, a member of the Stolen Generations, communicated the importance of family. For her, it was family that kept them strong while they were in the mission.

They tried to kill a lot of the kids, the spirit in “em but you know, …the love, or the something just kept us going you know, we knew we had families back in town, we knew we thought we were thousands of miles in the bush …. You know even though they tried to tear us away, a lot of the kids come back (i.e., to the reserve).”(Interview)

At the storytelling workshop she shared similar sentiments with the students, explaining that “They never killed our spirit you know, they never killed it. We knew when we were in there, they loved us, where our family was.” She reiterated this point at the end of her story, stating, “they can kill the body you know, but they can't kill the spirit *(laugh)* in all of us, you know *wadjelas* (i.e., white people) could put us down as much as they like, but somehow we always bounce back.” Individual and collective resilience was therefore linked to familial and social structures (Kirmayer et al., 2011).

The ongoing connectedness of Noongar people was evident in the Bush Baby storytelling and throughout fieldwork—people constantly emphasized how Noongar families in the town are all connected, and often spoke about the family history research they had done; an act of liberation. This sense of connection is epitomized by the excerpt from Enid, who commented, “we are all connected to the one group of descendants, we just split up like (*laugh*) the river… but it still flows with the one thing you know (*laugh*)” (Interview).

Collectively, these stories of cultural continuity and survival show that the narrators do not necessarily appropriate the stories they have been told of who they are. Instead, they highlight how people have created spaces and collective symbolic resources to deconstruct and speak back to dominant cultural narratives and maintain pride in and connection with their Aboriginal/Noongar identity, history, and culture.

## Discussion

In this article, we described a CACD project, Bush Babies, which was developed by CAN with Noongar communities in WA and was designed to support the desires of Noongar people to document their stories for current and future generations. The shared narratives, examined through critical constructionist narrative inquiry, shed light onto the deleterious impacts of colonial dispossession, assimilation, and racism, but also the ways in which individuals and communities have sought to protect themselves and hold onto valued aspects of identity and culture.

The Elders provided testimony of a history of dispossession that marks their lives and the legacy of this history. In doing so, they had an opportunity to name contemporary social realities (Sonn et al., 2013). The stories challenged deficit narratives that circulate in Australia about Noongar/Aboriginal people and culture by highlighting the historical roots of contemporary social issues facing communities. Specifically, they identified these issues as the legacy of past policies as well as ongoing experiences of racialization and exclusion. The Elders versions of history contest the pervasive willful ignorance of history and Aboriginal contributions that exists within Australia.

Through the spaces created by the Bush Babies project and the Rekindling strategy more broadly, Noongar people could voice histories of individual and collective suffering. Scholars highlighted the importance of remembering and voicing suffering as part of the process of healing from experienced trauma and struggles for social justice (Watkins & Shulman, 2008). As decolonial and liberation method, the telling of these stories is part of the process of digesting the past and its connection with the present, connecting the personal and the political, and in seeking healing and justice (Bell, 2010; Montero, 2009; Smith, 1999; Sonn et al., 2013). Those bearing witness to these stories are asked to acknowledge this history and its legacy in the present and, through hearing these stories, can better understand the connections between past and present realities.

In addition to remembering and counter‐storytelling, decolonization is also concerned with processes of cultural reclamation and renewal (Feeney, 2009; Zavala, 2016). Stories of resilience, resistance, and survival of people and culture play an important role in fostering pride in identity and belonging and processes of cultural reclamation and renewal. Resilience is conceptualized as residing in the resources people have available for narrating their lives (Kirmayer et al., 2011; Sonn & Fisher, 1998). In narrating cultural continuity and survival, the Elders pointed to the resources Noongar/Aboriginal people have for their own empowerment–symbolic resources that individuals and communities can be supported to (re)connect, (re)claim, strengthen and renew.

Recognizing and elevating these stories is central to challenging discourses of deficit and dysfunction and the preoccupation with woundedness (Lavallee & Clearsky, 2006) and victimhood, which epitomizes what Tuck (2009) described as “damage centered research.” This orientation also provides opportunities to recognize the various non‐pathological responses to the historical trauma experience, including expressions of resilience and survival (Denham, 2008). In telling these stories as part of a CACD project, the stories are put into the public record, acknowledged, and legitimized (Watkins & Shulman, 2008).

## Conclusion

Narrative approaches are important for showing the complex subjectivities and the constraints on subjectivities. Narrative inquiry allows for theorizing the possibilities for agency through the contestation and mobilization of symbolic resources. Researchers in community psychology and related disciplines can play a role in amplifying the voices of oppressed groups as part of the co‐intentional work of decolonization (Glover et al., 2010). It is important to provide resources to support Indigenous communities in their struggles for liberation, and their voices should be centered with the gaze directed at white supremacy or the coloniality of power (Lavallee & Clearsky, 2006). The Elders are the experts of their lives, and their stories are vital to processes of psychosocial transformation. Calls for decolonization and Indigenous approaches that center the humanity of self and other is vital to the goals of critical community psychology. As Maldonado‐Torres (2017) noted:

One's own humanity in turn, is expressed when one re‐claims the subjects and peoples that one encounters in the world and who live in precarious conditions. Reclamation of sub‐others is a decolonial attitude of “love and understanding” that is crucial in the task of epistemic and ontological decolonization. It is an epistemic (as well as ethical, social, and political) attitude that must not be subordinated to the desire for recognition, or to method.(p. 439)

By adopting a decolonizing standpoint (Reyes Cruz & Sonn, 2011), we can expand community psychology's ecology of knowledge by embracing diverse epistemologies, methodological pluralism, innovative methods, and relational ethics.

## Conflicts of Interest

None of the authors have any potential conflicts of interest related to this manuscript.

## References

[ajcp12367-bib-0001] Aboriginal and Torres Strait Islander Healing Foundation (2017). Bringing them home 20 years on: An action plan for healing. Available from: http://healingfoundation.org.au//app/uploads/2017/05/Bringing-Them-Home-20-years-on-FINAL-PRINT.pdf [last accessed June 15, 2019].

[ajcp12367-bib-0002] Archibald, L. (2006). Final report of the Aboriginal healing foundation volume III: Promising healing practices in Aboriginal communities. Ottawa, ON: Aboriginal Healing Foundation.

[ajcp12367-bib-0003] Atkinson, J. (2002). Trauma trails, recreating song lines: The transgenerational effects of trauma in Indigenous Australia. North Melbourne, Vic: Spinifex.

[ajcp12367-bib-0004] Australian Bureau of Statistics (2018). Estimates of Aboriginal and Torres Strait Islander Australians, June 2016 (cat no. 3238.0.55.001). Available from: http://www.abs.gov.au/ausstats/abs@.nsf/mf/3238.0.55.001 [last accessed June 15, 2019].

[ajcp12367-bib-0005] Australian Institute for Aboriginal and Torres Strait Islander Studies (2012). Guidelines for ethical research in Australian indigenous studies. Canberra, ACT: Australian Institute for Aboriginal and Torres Strait Islander Studies Available from: http://aiatsis.gov.au/research/ethical-research/guidelines-ethical-research-australian-indigenous-studies [last accessed June 15, 2019].

[ajcp12367-bib-0006] Australian Institute for Aboriginal and Torres Strait Islander Studies (2016). Mission and reserve records. Available from: https://aiatsis.gov.au/research/finding-your-family/family-history-sources/mission-and-reserve-records [last accessed June 15, 2019].

[ajcp12367-bib-0007] Australian Institute of Health and Welfare (2018). Aboriginal and torres strait islander stolen generations and descendants: Numbers, demographic characteristics and selected outcomes (cat no. IHW 195). Canberra, ACT: AIHW.

[ajcp12367-bib-0008] Bell, L. A. (2010). Storytelling for social justice: Connecting narrative and arts in antiracist teaching. New York, NY: Taylor & Francis.

[ajcp12367-bib-0009] Bennett, B. , Zubrzycki, J. , & Bacon, V. (2011). ‘What do we know?’ The experience of social workers working alongside Aboriginal people. Australian Social Work, 64, 20–37.

[ajcp12367-bib-0010] Bessarab, D. , & Ng'andu, B. (2010). Yarning about yarning as a legitimate method in indigenous research. International Journal of Critical Indigenous Studies, 3, 37–50.

[ajcp12367-bib-0011] Brave Heart, M. Y. H. (2003). The historical trauma response among natives and its relationship with substance abuse: A Lakota illustration. Journal of Psychoactive Drugs, 35, 7–13. 12733753 10.1080/02791072.2003.10399988

[ajcp12367-bib-0012] Chase, S. (2005). Narrative inquiry: Multiple lenses, approaches, voices In Denzin N. K. & Lincoln Y. S. (Eds.), The Sage handbook of qualitative research methods (3rd ed., pp. 651–680). Thousand Oaks, CA: Sage.

[ajcp12367-bib-0013] Collard, L. , Harben, S. , & , van den Berg, S. (2004). Nidja Beeliar Boodjar Noonookurt Nyininy: A Nyunger interpretive history of the use of Boodjar (country) in the vicinity of Murdoch University. Perth, WA: Murdoch University Available from: http://researchrepository.murdoch.edu.au/id/eprint/21353/1/Nidja_Beeliar_Boodjar_Noonookurt_Nyininy.pdf [last accessed June 15, 2019].

[ajcp12367-bib-0014] Community Arts Network Western Australia (2014). Annual report. Perth, WA: Community Arts Network Western Australia Retrieved from: http://www.canwa.com.au/about/annual-reports/.

[ajcp12367-bib-0015] Community Arts Network Western Australia (n.d.). Bush babies: Overview. Available from: http://www.canwa.com.au/project/bush-babies/ [last accessed June 15, 2019].

[ajcp12367-bib-0016] Crossley, M. (2007). Narrative analysis In Lyons E. & Coyle A. (Eds.), Analysing qualitative data in psychology (pp. 139–151). London, UK: Sage.

[ajcp12367-bib-0017] Denham, A. R. (2008). Rethinking historical trauma: Narratives of resilience. Transcultural Psychiatry, 45, 391–414. 18799640 10.1177/1363461508094673

[ajcp12367-bib-0018] Department of Regional Development (2014). Wheatbelt: A region in profile. Perth, WA: Government of Western Australia Available from: http://www.drd.wa.gov.au/Publications/Documents/A_region_in_profile_2014_Wheatbelt.pdf [last accessed June 15, 2019].

[ajcp12367-bib-0019] Dudgeon, P. , & Walker, R. (2015). Decolonising Australian psychology: Discourses. strategies, and practice. Journal of Social and Political Psychology, 3, 276–297.

[ajcp12367-bib-0020] Duran, E. , Duran, B. , Brave Heart, M. Y. H. , & Yellow Horse‐Davis, S. (1998). Healing the American Indian soul wound In Danieli Y. (Ed.), International handbook of multigenerational legacies of trauma (pp. 341–354). New York, NY: Plenum Press.

[ajcp12367-bib-0021] Emerson, P. , & Frosh, S. (2004). Critical narrative analysis in psychology: A guide to practice. Basingstoke, UK: Palgrave MacMillan.

[ajcp12367-bib-0022] Fanon, F. (2004). The wretched of the earth (R. Philcox trans.). New York, NY: Grove Press. (Original work published 1963).

[ajcp12367-bib-0023] Feeney, M. (2009). Reclaiming the spirit of well being: Promising healing practices for Aboriginal and Torres Strait Islander people. Canberra, ACT: The Stolen Generation Alliance.

[ajcp12367-bib-0024] Fine, M. (2014). Circuits of dispossession and privilege In Teo T. (Ed.), Encyclopaedia of critical psychology (pp. 227–234). New York, NY: Springer.

[ajcp12367-bib-0025] Frost, L. , & Hoggett, P. (2008). Human agency and social suffering. Critical Social Policy, 28, 438–460.

[ajcp12367-bib-0026] Glover, M. , Dudgeon, P. , & Huygens, I. (2010). Colonization and racism In Nelson G. & Prilleltensky I. (Eds.), Community psychology: In pursuit of liberation and well‐being (2nd ed., pp. 353–370). London, UK: Palgrave Macmillan.

[ajcp12367-bib-0027] Goldbard, A. (2006). New creative community: The arts of cultural development. Oakland, CA: New Village Press.

[ajcp12367-bib-0028] Gonzalez, J. , Simard, E. , Baker‐Demaray, T. , & Eyes, C. I. (2014). The internalized oppression of North American indigenous peoples In David E. J. R. (Ed.), Internalized oppression: The psychology of marginalized groups (pp. 31–56). New York, NY: Springer.

[ajcp12367-bib-0029] Government of Western Australia (2017). South west native title settlement. Available from: https://www.dpc.wa.gov.au/lantu/south-west-native-title-settlement/Pages/default.aspx [last accessed June 15, 2019].

[ajcp12367-bib-0030] Haebich, A. (2000). Broken circles: Fragmenting Indigenous families: 1800–2000. Fremantle, WA: Fremantle Arts Centre Press.

[ajcp12367-bib-0031] Human Rights and Equal Opportunity Commission (1997). Bringing them home: National inquiry into the separation of aboriginal and torres strait Islander children from their families. Canberra, ACT: Commonwealth of Australia.

[ajcp12367-bib-0032] Kickett‐Tucker C., Bessarib D., Coffin J., & Wright M. (Eds.) (2017). Aboriginal community development: Fostering cultural security. Cambridge, UK: Cambridge University Press.

[ajcp12367-bib-0033] Kirmayer, L. J. , Dandeneau, S. , Marshall, E. , Phillips, M. K. , & Williamson, K. J. (2011). Rethinking resilience from indigenous perspectives. The Canadian Journal of Psychiatry, 56, 84–91. 21333035 10.1177/070674371105600203

[ajcp12367-bib-0034] Kovach, M. E. (2009). Indigenous methodologies: Characteristics, conversations, and contexts. Toronto, ON: University of Toronto Press.

[ajcp12367-bib-0035] Krieg, A. (2009). The experience of collective trauma in Australian Indigenous communities. Australasian Psychiatry, 17, 28–32. 10.1080/10398560902948621 19579102

[ajcp12367-bib-0036] Lavallee, B. , & Clearsky, L. (2006). ‘From woundedness to resilience’: A critical review from an Aboriginal perspective. International Journal of Indigenous Health, 3, 4–6.

[ajcp12367-bib-0037] Lyons, E. (2007). Analysing qualitative data: Comparative reflections In Lyons E. & Coyle A. (Eds.), Analysing qualitative data in psychology (pp. 164–177). London, UK: Sage.

[ajcp12367-bib-0038] Maldonado‐Torres, N. (2017). Frantz Fanon and the decolonial turn in psychology: From the modern/colonial methods to the decolonial attitude. South African Journal of Psychology, 47, 432–441.

[ajcp12367-bib-0039] Miles, M. B. , & Huberman, A. M. (1994). Qualitative data analysis: An expanded sourcebook (2nd ed.). Thousand Oaks, CA: Sage.

[ajcp12367-bib-0040] Moane, G. (2011). Gender and colonialism: A psychological analysis of oppression and liberation (2nd ed.). London, UK: Palgrave MacMillan.

[ajcp12367-bib-0041] Montero, M. (2009). Methods for liberation: Critical consciousness in action In Montero M. & Sonn C. (Eds.), Psychology of liberation. Theory and applications (pp. 73–92). New York: Springer.

[ajcp12367-bib-0042] Montero, M. , Sonn, C. C. , & Burton, M. (2017). Community psychology and liberation psychology: A creative synergy for an ethical and transformative praxis In Bond M. A., Serrano‐Garcia I. & Key C. B. (Eds.), APA handbook of community psychology, vol 1 (pp. 149–167). Washington, DC: American Psychological Society.

[ajcp12367-bib-0043] Quayle, A. (2017). Narrating oppression, psychosocial suffering and survival through the Bush Babies Project. (Unpublished doctoral dissertation). Victoria University, Melbourne, Vic..

[ajcp12367-bib-0044] Quayle, A. , Sonn, C. C. , & van den Eynde, J. (2016). Narrating the accumulation of dispossession: Stories of Aboriginal Elders. Community Psychology in Global Perspective, 2, 79–96.

[ajcp12367-bib-0045] Rappaport, J. (2000). Community narratives: Tales of terror and joy. American Journal of Community Psychology, 28, 1–24. 10824272 10.1023/a:1005161528817

[ajcp12367-bib-0046] Reyes Cruz, M. , & Sonn, C. C. (2011). (De)colonizing culture in community psychology: Reflections from critical social science. American Journal of Community Psychology, 47, 203–214. 21052821 10.1007/s10464-010-9378-x

[ajcp12367-bib-0047] Santos, B. D. S. (2014). Epistemologies of the south: Justice against epistemicide. New York, NY: Routledge.

[ajcp12367-bib-0048] Smith, L. T. (1999). Decolonizing methodologies: Research and indigenous peoples. London, UK: Zed Books.

[ajcp12367-bib-0049] Smith, B. , & Sparkes, A. C. (2008). Contrasting perspectives on narrating selves and identities: An invitation to dialogue. Qualitative Research, 8, 5–35.

[ajcp12367-bib-0050] Solórzano, D. G. , & Yosso, T. J. (2002). Critical race methodology: Counter‐storytelling as an analytical framework for education research. Qualitative Inquiry, 8, 23–44.

[ajcp12367-bib-0051] Sonn, C. C. , & Fisher, A. T. (1998). Sense of community: Community resilient responses to oppression and change. Journal of Community Psychology, 26, 457–471.

[ajcp12367-bib-0052] Sonn, C. C. , Stevens, G. , & Duncan, N. (2013). Decolonisation, critical methodologies and why stories matter In Stevens G., Duncan N. & Hook D. (Eds.), Race, memory and the apartheid archive: Towards a transformative psychosocial praxis (pp. 295–314). New York, NY: Palgrave Macmillan.

[ajcp12367-bib-0053] Sparkes, A. C. , & Smith, B. (2013). Narrative constructionist inquiry In Holstein J. A. & Gubrium J. F. (Eds.), Handbook of constructionist research (pp. 295–314). London, UK: The Guildford Press.

[ajcp12367-bib-0054] State Library of Western Australia (2016). Storylines. Available from: http://slwa.wa.gov.au/for/indigenous_australians/storylines [last accessed June 15, 2019].

[ajcp12367-bib-0055] Tuck, E. (2009). Suspending damage: A letter to communities. Harvard Educational Review, 79, 409–428.

[ajcp12367-bib-0056] Vizenor G. (Ed.) (2008). Survivance: Narratives of native presence. Lincoln, NE: University of Nebraska Press.

[ajcp12367-bib-0057] Walker, R. , Schultz, C. , & Sonn, C. (2014). Cultural competence: Transforming policy, services, programs and practice In Dudgeon P., Milroy H. & Walker R. (Eds.), Working together: Aboriginal and Torres Strait Islander mental health and wellbeing principles and practice (2nd ed., pp. 195–220). Barton, ACT: Commonwealth of Australia.

[ajcp12367-bib-0058] Watkins, M. , & Shulman, H. (2008). Toward psychologies of liberation. Basingstoke, UK: Palgrave MacMillan 10.1057/9780230227736.

[ajcp12367-bib-0059] Zavala, M. (2016). Decolonial methodologies in education In Peters M. (Ed.), Encyclopedia of educational philosophy and theory (pp. 361–366). Singapore: Springer 10.1007/978-981-287-532-7.

